# Does sucrose intake affect antropometric variables, glycemia, lipemia and C-reactive protein in subjects with type 1 diabetes?: a controlled-trial

**DOI:** 10.1186/1758-5996-5-67

**Published:** 2013-11-12

**Authors:** Débora Lopes Souto, Lenita Zajdenverg, Melanie Rodacki, Eliane Lopes Rosado

**Affiliations:** 1 Institute of Nutrition Josué de Castro, Federal University of Rio de Janeiro, Brigadeiro Trompowski avenue, CCS building, - J block J - second floor - District: Ilha do Fundão, 360 Felisbelo Freire Street, Apartament 202 District: Ramos, Zipe Code: 21941-590, Rio de Janeiro, Brazil; 2 Medicine, Federal University of Rio de Janeiro, Rio de Janeiro, Brazil; 3 Food Science and Technology, Federal University of Viçosa, Viçosa, Brazil; 4 Institute of Nutrition Josué de Castro, Federal University of Rio de Janeiro, Rio de Janeiro, Brazil; 5 Department of Internal Medicine, Section of Diabetes and Nutrology, Federal University of Rio de Janeiro, Rio de Janeiro, Brazil

**Keywords:** Diabetes, Sucrose, Body composition, C-reactive protein, Carbohydrate

## Abstract

**Background:**

It is unclear if the sugar intake may affect metabolic parameters in individuals with type 1 diabetes. Therefore, the purpose of this study was to evaluate the effects of sucrose intake in glycemic, lipemic, anthropometric variables, as well as in C-reactive protein (CRP) levels in these individuals.

**Methods:**

Thirty-three subjects with type 1 diabetes were evaluated at baseline and 3-months after intervention. Volunteers were randomized into groups: sucrose-free (diet without sucrose) or sucrose-added (foods containing sucrose in composition). Both groups received the same macronutrient composition and used the carbohydrate counting methods. All underwent an interview and anthropometric evaluation. Blood was drawn for glycated haemoglobin, glucose, total cholesterol, HDL, and CRP measurement, and the medical charts were reviewed in all cases.

**Results:**

At baseline, anthropometric, clinical and laboratory variables did not differ between groups, except for the triglycerides. Although at baseline triglycerides levels were higher in the sucrose-added group (p = 0.01), they did not differ between groups after the intervention (p = 0.92). After 3-months, CRP was higher in the sucrose-added than in the sucrose-free group (p = 0.04), but no further differences were found between the groups, including the insulin requirements, anthropometric variables, body composition, and glycemic control. Both groups showed sugars intake above the recommendations at baseline and after intervention.

**Conclusions:**

Sucrose intake, along with a disciplined diet, did not affect insulin requirements, anthropometric variables, body composition, lipemic and glycemic control. However, although the sucrose intakes increase CRP levels, the amount of sugar in the diet was not associated with this inflammatory marker.

## Background

Sucrose is a very attractive source of carbohydrate [1]. The preference for sucrose may be influenced by genetic factors [2-5], and others complex behaviors (such as craving, infant exposure, social habits, and personal dietary choices) [6-10]. The effect of sugars on lipid metabolism remains an extremely active area of inquiry because has been shown that high-sugar diets may increase triglycerides levels in subjects with type 2 diabetes [11-13], but they do not seem to affect the lipid profile in subjects with type 1 diabetes, if optimal glycemic control is preserved [14-18].

Carbohydrate is the major determinant of postprandial glucose levels. The carbohydrate counting is the best method for estimating the grams of carbohydrates in a meal and then calculating the pre-meal insulin dose based on the self-monitored blood glucose (SMBG) and insulin-to-carbohydrate ratio [1,19].

The American Diabetes Association nutrition recommendations state that the meal plans based on carbohydrate counting remains a key strategy to achieve the glycemic control [1] because the adjustment of pre-prandial insulin doses to the amounts of dietary carbohydrates ingested during the subsequent meal resulted in improved in glycemic control [20-24], self-management skills, quality of life, and dietary freedom [25-29].

However, the basic and advanced carbohydrate counting are the common methods used currently in clinical practice [19,22,30]. In the basic method, the subjects are encouraged to eat constant amounts of carbohydrate at meals. This is useful to understand the effect of food, insulin and to identify the portion sizes, considering that one carbohydrate serving have an approximately 15 g of carbohydrates (these information are obtained from exchange lists, internet and from the nutrition facts). In the advanced method, the patients should have a good understanding of carbohydrate counting principles, as well as understanding pattern management and how to use insulin-to-carbohydrate ratios [1,19,30]. According to described, the inclusion of sucrose in the dietary plan of individuals with type 1 diabetes is quite appealing and has been a focus of interest, especially after the introduction of the carbohydrate counting methods. However, previous studies suggested that sugar intake may active inflammation pathways and increase circulatory levels of the inflammatory markers, such as C-reactive protein (CRP) both in health individuals [31-33] and patients with type 2 diabetes [34]. However, clinical trials in type 1 diabetes are still lacking.

The goal of this study was to investigate the influence of sucrose intake on anthropometric variables, body composition, lipemia, glycemic control and CRP levels in subjects with type 1 diabetes.

## Subjects and methods

This is a controlled clinical-trial was conducted between July 2009, and January 2011. Participants with type 1 diabetes (disease duration of 24 years or more) were recruited at the waiting room of the Clementino Fraga Filho University Hospital, Brazil. Patients with body mass index (BMI) ≥ 30 kg/m^2^, smokers, alcoholics, users of lipid-lowering or oral hypoglycemic medications and other diseases (such as hypertension, celiac disease, hypo- and hyperthyroidism) were not included.

The hospital database update on January 2010, the size of the universe is 200 outpatients. Of these, only 80 (40%) of these cases were eligible and were then contacted and invited to participate. Forty-five (22%) refused and 35 (17.5%) volunteers agreed to participate in the study. All signed an informed consent and the protocol was approved by the Ethical Committee (Institutional Review Board, protocol 050/09). During the follow-up, two patients were excluded (one had infection and another because did not use insulin properly) and a total of 33 (16.5%) participants completed the study. The sample is not representative and was selected for convenience, thus, results are not intended to represent exactly what would happen with a population [35].

All volunteers were assessed at baseline and after 3-months of intervention. They received three individual face-to-face consultation sessions which included advices on food purchased, food selection, portion sizes, cooking methods, and effect of food on glycemic control.

Participants were allocated into two groups, according to their sucrose intake reported in three 24-hour recalls. Individualized diet prescription based on the current recommendations (dietary energy content of 50-60% carbohydrates, 15-20% of protein, 25-35% of total fat, less than 7% of saturated fatty acids, a maximum of 10% 10% from polyunsaturated fatty acids, and 10-15% of monounsaturated fatty acids) [1].

Percent energy from macronutrients was similar in both groups [1,36] and as well as the same instructions about carbohydrate counting, and exchange lists with sucrose-free or with foods containing sucrose in its compositions (for sucrose-added group). The lists have been developed based on “*Choose your foods: Exchange lists for diabetes*” [37] and contained more than 200 foods with a similar amount of carbohydrates (approximately 15 g of carbohydrates per serving), however we detailed listings in the catalogues of permitted and prohibited foods, based on the amount of sugar in each product were also provided to the participants. Diets and dietary records were analyzed using Software DietPró 5.5i (version 2008–2011) and the cutoff point for sucrose intake was < 7 or ≥ 7% to sucrose-free and sucrose-added group, respectively.

The carbohydrate counting method was selected as according to ability to the patient’s understand the management plan and how to use insulin-to-carbohydrate ratios properly [1,19]. The basic and advanced carbohydrate counting were equally distributed between groups (p = 0.62). Basic method was used for 50% (n = 9) in sucrose-free and 53.33% (n = 8) in sucrose-added group, and the advanced method was used for 50 and 46.67% (n = 9 for each) of volunteers in sucrose-free and sucrose-added group, respectively.

Baseline dietary intake was evaluated from 3-day diet records. Volunteers were followed monthly when 24-hour recalls were performed to verify adherence to the diet. Additionally, they were followed once a week by telephone calls [38].

Insulin, glucometer and test strips to check their SMBG four-daily were provided to all participants. The insulin sensitivity factor was calculated as 1800 or 1500 (for rapid insulin analogs and regular insulin, respectively) divided by the total daily insulin dose. Insulin-to-carbohydrate ratios were calculated as 500 or 450 divided by the total daily insulin dose (for rapid insulin analogs and regular insulin, respectively) and frequently were adjusted 2-hour postprandial. Patients were instructed to calculate their premeal insulin bolus doses based on carbohydrate intake, individualized insulin-to-carbohydrate ratios, and theirs SMBG [39].

Blood sample were obtained after eight hours fasting, and events that could influence the results were considered (such as: infections, flu, fever). Glycated haemoglobin was performed by high-performance liquid chromatography [40]. Fasting glucose, total cholesterol, HDL and triglycerides were measured by enzymatic colorimetric method, and CRP was determined by ultrasensitive colorimetric enzyme-linked immunosorbent assay [41]. LDL cholesterol was calculated with the Friedewald equation [42].

Body mass index was calculated as body weight in kilograms divided by the square of height in meters [43]. Waist circumference was determined as the average of two measurements calculated to the nearest 0.1 cm midway between the lower rib margin and the iliac crest after a normal expiration [44]. Body composition was measured by tetrapolar bioelectrical impedance (biodynamic Model 450) [45].

Statistical analyzes were performed in SPSS software (version 16.0; SPSS Inc, Chicago, IL) with significance level of 5%. Quantitative variables were described as the mean and standard deviation. Mann–Whitney test was used for between-group comparison and Wilcoxon test to compare the effects of nutrition-knowledge in each group. Linear regression was used to determine the value of the triglycerides and CRP levels based upon the values of other variables.

## Results

Thirty three patients with type 1 diabetes (21 men and 12 women) with a mean age of 21.7 ± 5 years old (range, 15 to 37) and mean duration of disease of 11.9 ± 6.4 years (range, 2 to 18) were included (Table 1). All were in a basal-bolus plan, 32 using multiple daily injections and one in insulin-pump.

**Table 1:** **Anthropometric characteristics at baseline and after intervention in sucrose-free and sucrose-added groups**^**♦**^

	**Baseline**			**After intervention**			**p value**^**║**^	**p value**^**#**^
	**Sucrose-free**	**Sucrose-added**	**p value**^**‡**^	**Sucrose-free**	**Sucrose-added**	**p value**^**‡**^		
	**(n = 18)**	**(n = 15)**		**(n = 18)**	**(n = 15)**			
M/F (n)	12/6	8/7	0.53	12/6	8/7	0.53	-	-
BMI (kg/m^2^)	22.40 ± 2.65	24.02 ± 2.56	0.07	22.84 ± 2.51	23.89 ± 2.39	0.19	0.23	0.34
WC (cm)	76.50 ± 7.78	76.60 ± 7.70	0.85	76.22 ± 7.83	76.40 ± 7.16	0.92	0.51	0.48
Body fat (%)	18.57 ± 7.05	23.64 ± 8.76	0.05	18.81 ± 6.94	22.94 ± 9.51	0.14	0.64	0.47
LBM (%)	81.44 ± 7.05	76.34 ± 8.77	0.05	81.18 ± 6.94	76.72 ± 9.14	0.07	0.73	0.52
TBW (%)	38.29 ± 6.50	37.50 ± 8.97	0.60	38.52 ± 5.62	38.44 ± 9.01	0.53	0.39	0.18

^♦^Data are mean ± SD.

^‡^*p-values* were derived by *Mann–Whitney* test.

^║^*p-values* were derived by analysis of covariance with basal and values during the nutritional intervention in sucrose-free group (*Wilcoxon* signed rank test).

^#^p values were derived by analysis of covariance with basal and values during and after the nutritional intervention in sucrose-added group (*Wilcoxon* signed rank test).

Legend/abbreviations: *BMI* Body mass index, *LBM* Lean body mass, *M/F* Male/female, *TBW* Total body water, *WC* Waist circumference.

Ten patients used the carbohydrate counting method prior to the study (30%), and this proportion did not differ between groups (p = 0.33).

Anthropometric, biochemical and clinical basal characteristics were similar between groups, except for the triglycerides levels, that were higher in the sucrose-added group (p = 0.01) (Tables 1 and 2). Nevertheless, regression analysis showed no association between triglycerides and other variables (p > 0.05).

**Table 2:** **Biochemical and clinical characteristics at baseline and after intervention in sucrose-free and sucrose-added groups**^**♦**^

	**Baseline**			**After intervention**			**p value**^**║**^	**p value**^**#**^
	**Sucrose-free**	**Sucrose-added**	**p value**^**‡**^	**Sucrose-free**	**Sucrose-added**	**p value**^**‡**^		
	**(n = 18)**	**(n = 15)**		**(n = 18)**	**(n = 15)**			
Glucose (mmol/L)	9.42 ± 3.86	10.86 ± 3.61	0.25	9.46 ± 4.18	8.63 ± 5.1	0.40	0.87	0.24
HbA1c (%)	7.33 ± 1.06	8.02 ± 2.14	0.57	7.28 ± 0.91	7.76 ± 1.53	0.65	0.67	0.67
TC (mmol/L)	8.59 ± 1.84	9.36 ± 2.44	0.32	8.70 ± 1.89	9.61 ± 3.22	0.42	0.98	0.69
HDL (mmol/L)	2.72 ± 0.65	2.92 ± 0.78	0.32	2.67 ± 0.60	2.94 ± 0.78	0.23	0.37	0.75
LDL (mmol/L)	4.77 ± 1.40	5.14 ± 1.80	0.46	4.69 ± 1.53	5.27 ± 2.10	0.34	0.44	0.84
TG (mmol/L)	3.48 ± 1.43	5.36 ± 2.33	0.01	4.18 ± 1.44	5.65 ± 4.58	0.92	0.06	0.79
CRP (mmol/L)	0.27 ± 0.20	0.38 ± 0.20	0.17	0.29 ± 0.13	0.42 ± 0.24	0.04	0.79	0.94
TBD (UI/kg/d)	0.69 ± 0.17	0.61 ± 0.19	0.19	0.68 ± 0.16	0.61 ± 0.19	0.27	1.00	1.00
ICR	14.45 ± 5.12	11.84 ± 1.88	0.11	14.45 ± 5.12	11.84 ± 1.88	0.11	1.00	1.00
CF	35.88 ± 7.12	40.00 ± 8.45	0.10	35.88 ± 7.12	40.00 ± 8.45	0.10	1.00	1.00
SMBG (mmol/L)	8.32 ± 1.43	9.02 ± 1.91	0.31	8.32 ± 1.43	9.02 ± 1.91	0.31	1.00	1.00
MDI (times/day)	3.70 ± 0.84	3.73 ± 0.70	0.79	3.70 ± 0.84	3.73 ± 0.70	0.79	1.00	1.00

^♦^Data are mean ± SD.

^‡^*p-values* were derived by *Mann–Whitney* test.

^║^*p-values* were derived by analysis of covariance with basal and values during the nutritional intervention in sucrose-free group (*Wilcoxon* signed rank test).

^#^p values were derived by analysis of covariance with basal and values during and after the nutritional intervention in sucrose-added group (*Wilcoxon* signed rank test).

Legend/abbreviations: *CF* Correction factor, *CRP* C-reactive protein, *HbA1c* Glycosylated hemoglobin, *ICR* Insulin-to-carbohydrate ratio, *MDI* Multiple daily injections using fast-acting insulin, *SMBG* Self-monitored blood glucose, *TBD* Total basal dose, *TC* total cholesterol, *TG* triglycerides.

Anthropometric variables were not associated with insulin sensitivity factor, total daily insulin dose or insulin-to-carbohydrate ratio (p > 0.05).

Both groups had a hypocaloric, hyperprotein, normoglycidic, normolipidic and an adequate fiber intake, when compared with the American Diabetes Association [1] and Dietary Reference Intakes [36] current recommendations. There were no differences between groups in these parameters. Sucrose (p = 0.01) and saturated fatty acids (p = 0.03) intake were higher in the sucrose-added than in the sucrose-free group, however, both groups showed simple carbohydrate and saturated fatty acids intakes above the daily recommended intake based on the current guidelines [1,36] (Table 3).

**Table 3:** **Recommended dietary allowance and actual dietary intake at baseline and after intervention in sucrose-free and sucrose-added groups**^**♦**^

	**Baseline**			**After intervention**			**p value**^**║**^	**p value**^**#**^
	**Sucrose-free**	**Sucrose-added**	**p value**^**‡**^	**Sucrose-free**	**Sucrose-added**	**p value**^**‡**^		
	**(n = 18)**	**(n = 15)**		**(n = 18)**	**(n = 15)**			
Recommended dietary allowance (kcal)	2589.66 ± 441.01	2539.90 ± 370.38	0.60	2589.66 ± 441.01	2539.90 ± 370.38	0.60	-	-
Energy (kcal)	2117.29 ± 454.99	2158.58 ± 504.65	0.66	2332.93 ± 309.20	2367.71 ± 426.28	0.40	0.01	0.02
Carbohydrate (%)	51.32 ± 6.90	50.27 ± 6.07	0.80	53.19 ± 5.89	58.42 ± 4.57	0.57	0.28	0.00
Sucrose (%)	5.29 ± 6.59	17.21 ± 14.12	0.01	2.34 ± 1.16	27.32 ± 13.47	0.00	0.03	0.06
Fructose (%)	6.66 ± 5.98	7.78 ± 7.51	0.77	5.98 ± 4.14	9.43 ± 7.85	0.31	0.68	0.30
Protein (%)	18.38 ± 2.68	19.25 ± 3.15	0.56	19.80 ± 2.53	19.88 ± 3.37	0.89	0.12	0.17
Fat (%)	29.43 ± 6.78	30.14 ± 7.24	0.85	24.22 ± 7.96	25.66 ± 4.53	0.49	0.07	0.03
Saturated fatty acids (%)	9.25 ± 3.62	11.44 ± 4.24	0.03	8.67 ± 3.38	9.93 ± 3.63	0.38	0.81	0.10
Polyunsaturated fatty acids (%)	5.89 ± 3.45	5.18 ± 2.80	0.36	3.66 ± 2.30	4.13 ± 1.48	0.12	0.00	0.21
Monounsaturated fatty acids (%)	7.78 ± 2.99	7.40 ± 1.84	0.75	4.25 ± 2.52	6.60 ± 1.49	0.00	0.00	0.15
Fiber intake (g)	24.77 ± 8.11	25.86 ± 13.78	0.82	37.48 ± 13.08	34.44 ± 13.99	0.69	0.00	0.02

^♦^Data are mean ± SD; ^‡^*p-values* were derived by *Mann–Whitney* test.

^║^*p-values* were derived by analysis of covariance with basal and values during the nutritional intervention in sucrose-free group (*Wilcoxon* signed rank test).

^#^p values were derived by analysis of covariance with basal and values during and after the nutritional intervention in sucrose-added group (*Wilcoxon* signed rank test).

### Characteristics of groups after intervention

During the intervention, both groups remained in a hypocaloric, hyperprotein, normoglycidic, normolipidic and adequate fiber diet [1,36]. The sucrose-added group continued to show a higher sucrose intake than sucrose-free group (p < 0.01), however both groups presented simple carbohydrate intake below the recommendations [1,36]. The monounsaturated fatty acids intake was higher in the sucrose-added group, when compared to the sucrose-free group (p < 0.01), but both groups presented intakes below the recommendations [1,36]. The other nutrients did not differ between groups (Table 3).

Comparing the intake before and after intervention, the sucrose-free group increased the sucrose, polyunsaturated and monounsaturated fatty acids intake, however reduced the energy and fiber intake (p < 0.05). The Sucrose-added group reduced energy, carbohydrate and fiber, while increased fat intake (p < 0.05) (Table 3).

The data included an average of 111 ± 15.57 SMBG per month for each participant. Insulin requirements, anthropometric and laboratory variables did not differ after the intervention, in any of the groups. The sucrose-added group presented higher CRP concentrations than others volunteers (p = 0.04), although other variables did not differ between groups (p > 0.05). CRP was not associated with any of the anthropometric or laboratory variables (p > 0.05). Regression analysis showed no association between the amount of sucrose intake and CRP levels in any of the groups (p > 0.05) (Table 2).

Triglycerides showed a positively association with the mean of SMBG levels (r = 0.71; p = 0.00) only in the sucrose-added group, however, did not differ between groups after intervention (p = 0.92), as opposed to baseline (p = 0.01). There was a trend towards an increase in triglycerides levels in the sucrose-free group (p = 0.06), which was not observed in the sucrose-added group (p = 0.79) (Table 2). However, the amount of sucrose intake was positively associated to triglyceride levels (r = 0.52; p = 0.04).

## Discussion

In this study we showed that the sugar intake did not affect the anthropometric variables, body composition and glycemic control after 3-months in subjects with type 1 diabetes. This was the first clinical trial to assess the influence of sucrose in these variables in individuals with type 1 diabetes and showed a link between sucrose intake and increase of CRP. Studies have reported that CRP levels are higher in subjects with type 1 [46] and type 2 diabetes [47] compared with those without diabetes, while another study not find any correlation between CRP levels and titer of autoantibodies in long-term type 1 individuals with type 1 diabetes [48].

Previous studies have shown positive correlation between sugar intake and CRP in rats [49,50], healthy adults [51-54], children [55], obese [32,56], and individuals with type 2 diabetes [57-59], suggesting several possible mechanisms. One explanation would be the stimulation of the inflammatory response as a consequence of hyperglycemia [53,57,60]. Another mechanism could be an effect of glucose and fructose in enzymatic pathways and in the transcription factors involved in lipogenesis. This could lead to peroxisome proliferation changes, microsomal enzyme induction, and transcription of inflammatory factors by the activating nuclear factor-κB [61-63]. Alternatively, our third hypothesis is that the chronic hyperglycemia combined with sugar intake could induced release of the neuropeptide Y (a sympathetic neurotransmitter) directly into the adipose tissue, which stimulates endothelial cell (angiogenesis), and consequently leads to increase cytokines and acute phase proteins [64].

There are other hypotheses to explain why high sugars intake could lead to an increase in the levels of inflammatory markers. However, these mechanisms have been observed in mice [61,62], healthy [53,63], obese [63,65] or individuals with type 2 [57,63]. To our knowledge, this was the first study to examine the effect of sucrose intake in the CRP levels in the subjects with type 1 diabetes.

Even though the between-group differences in CRP in the present study were small, and CRP has been used as a consistent marker for evaluating the extent of cardiovascular diseases in subjects with type 1 diabetes [66-70], we suggest that others determinants, such as genetic predisposition, coping mechanisms, and environmental factors, make individuals more susceptible to changes in this inflammatory marker. Therefore, further studies are necessary to understand the effect of sugar intake in CRP levels.

In addition, we showed that triglycerides levels did not differ between groups after intervention, in contrast to baseline, suggesting a trend toward an increased in triglycerides levels in sucrose-free group. Although, the increase in triglyceride levels was not statistically significant, a possible reason for this worsening might be the reduced fiber intake [71,72].

Furthermore, the scarcity of controlled studies assessing the effect of sucrose intake on metabolic control in well controlled subjects with type 1 diabetes difficult the comparison with other studies. Controlled studies assessing the effect of sucrose in metabolic control of individuals with type 1 diabetes are still lacking because the studies have used fructose as sugar source [73-75] or high-glycemic index diet [15,76,77]. Only one observational study reported an association between sugar-sweetened beverages and high triglycerides levels in subjects with type 1 diabetes [78], while a controlled trial showed no effect of foods with sucrose on triglycerides levels in this population [18]. Therefore, although our data suggests that the sucrose intake did not change triglycerides levels, further larger and longer studies are still necessary to elucidate this finding.

Furthermore, in this study, triglycerides had no relationship with CRP. This finding is opposite to other studies, which suggest that strategies to decrease inflammatory activity in type 1 diabetes should focus on the triglycerides levels [79]. Corroborating previous studies that have associated the glycemic control with triglycerides, we observed a positively association between triglycerides and SMBG, demonstrating the glycemic control is an important mediator of lipid abnormalities [16,17,80]. This may occur because the insulin influences the activity of lipase lipoprotein and inhibits the lipolysis of fats stored in the tissues by inhibition of hormone-sensitive lipase. Thus, such as endogenous insulin, the effective insulin treatments influence the lipid transfers in well-controlled patients with type 1 diabetes [16].

There are potential limitations regarding the interpretation of our data. Firstly, two characteristic (triglycerides levels and saturated fatty acids intake) differed between groups at baseline. In addition, the sucrose intake was not the only dietary factor that differed between groups during the intervention (sucrose-added group presented a higher monounsaturated fatty acids intake than the sucrose-free group). Interestingly, both groups had fatty acids intakes above the current recommendations, based on the American Diabetes Association guidelines [1]. The first and second limitations probably occurred because the sample was selected by convenience [35]. Furthermore, the adherence to the prescribed diet is difficult to accomplish [81-84]. Thus, these results could not represent what would happen with the entire population [35].

In summary, although American Diabetes Association report that “*unnecessarily restrict sucrose*” [1] and all our subjects (both groups) had less than 10% of energy from sugars, we showed that intake of sucrose did not alter body weight, body composition, glycemic and lipemic control, however, there is a link between sucrose intake and increase of CRP. For this reason, according to the above result, we suggest that individuals with diabetes choose to avoid high-sucrose foods even they may eat a relatively small amount. Therefore, further clinical studies are needed to assess the relationship between sugars and CRP levels in subjects with type 1 diabetes.

## Conclusions

Sucrose intake, along with a disciplined diet, compared with sucrose-free diet, did not affect insulin requirements, anthropometric variables, body composition, glycemic, and lipemic control. However, although the sucrose intakes increase CRP levels, the amount of sugar in the diet was not associated with this inflammatory marker.

## Abbreviations

BMI: Body mass index; CRP: C-reactive protein; SMBG: Self-monitored blood glucose.

## Competing interests

The authors declare that they have no competing interests.

## Authors’ contributions

DLS draft the manuscript, conceived, performed and coordinated the study. LZ and MR helped in the data collecting and contributed to draft the manuscript. ELR participated in design and coordination and draft the manuscript. All authors read and approved the final manuscript.
